# Linking community pharmacy dispensing data to prescribing data of general practitioners

**DOI:** 10.1186/1472-6947-6-18

**Published:** 2006-04-03

**Authors:** Stefan R Florentinus, Patrick C Souverein, Fabiënne AMG Griens, Peter P Groenewegen, Hubert GM Leufkens, Eibert R Heerdink

**Affiliations:** 1 Utrecht University; Faculty of Pharmaceutical Sciences, Department of Pharmacoepidemiology and Pharmacotherapy, Utrecht Institute for Pharmaceutical Sciences (UIPS), Utrecht, The Netherlands; 2 NIVEL (Netherlands Institute for Health Services Research), Utrecht, The Netherlands; 3 Utrecht University, Faculty of Social Sciences and Faculty of Geosciences, Department of Sociology and Department of Human Geography, Utrecht, the Netherlands; 4 Foundation for Pharmaceutical Statistics, The Hague, The Netherlands

## Abstract

**Background:**

Databases are frequently used for pharmacoepidemiological research. However, most of these databases consist either of prescribing, dispensing or administrative data and therefore lack insight in the interaction between the several health professionals around the patient.

**Methods:**

To determine the success rate of linking records from the dispensing database of the Foundation for Pharmaceutical Statistics to the prescribing database of the second Dutch national survey of general practice, conducted by NIVEL (Netherlands Institute for Health Services Research), a deterministic record linkage approach was used with patient and prescription characteristics as matching variables between the two databases.

**Results:**

The catchment area included 123 community pharmacies, 90 GP practices and approximately 170,000 unique patients. Overall 110,102 (64.8%) unique patients were linked using the matching variables patient's gender, year of birth, the 4-digit part of the postal code, date of dispensing/prescribing and ATC-code. The final database contains of the 110,102 both prescribing data from 83 GP practices and dispensing data of 112 community pharmacies.

**Conclusion:**

This study shows that linkage of dispensing to prescribing data is feasible with a combination of patient characteristics, such as gender, year of birth and postal code, and prescription characteristics like prescription date and ATC-code. We obtained a linkage proportion of 64.8% resulting in complete prescribing and dispensing history of 110,102 patients. This offers an opportunity to gain insight in the mechanisms and factors influencing drug utilisation in general practice.

## Background

To enhance the quality and efficiency of observational research in daily clinical practice, linkage of different databases is often desirable to gain more insight in the underlying mechanisms by which medicines are utilised in large populations. Linkage of dispensing data from community pharmacies to hospital admission data, for example, has proven to be beneficial in detecting serious drug-induced side-effects [1,2]. Although important findings have been published, most of the databases used so far consist of either prescribing data of general practitioners (GPs), pharmacy records or administrative data from health insurance companies [3,4]. The origin of these databases defines the vantage point of the researcher and may therefore limit the usefulness of the data. For example, prescribing data of GPs alone are not sufficient to compile a clear overview of all drugs prescribed in general practice. Prescriptions of hospital specialists are usually not archived in GP prescribing databases and, unless repeated by GPs, result in blind spots. Furthermore, due to interventions of community pharmacists to optimise pharmacotherapy or prevent possible side effects the dispensed drug may not be the same as the drug prescribed by the GP. This can also cause differences in prescribing and dispensing databases [5].

In the Netherlands, several organisations are involved in routine collection of medical records, such as prescribing data of GPs [6,7] and dispensing data from community pharmacies [8]. In 2000, NIVEL (Netherlands Institute for Health Services Research) launched the second Dutch national survey of general practice (DNSGP-2) to obtain up-to-date and national representative data on the Dutch general practice. The rationale and design of the DNSGP-2 is discussed in detail elsewhere [9,10]. The DNSGP-2 resulted among other things in a dataset comprising over two million prescriptions, prescribed by 101 GP practices to 262,817 patients in the period October 2000 to January 2002. For each prescription information was available on the prescription date, quantity prescribed, duration of use, product code, ATC code, [11] and ICPC coded diagnose [12]. GPs were asked to fill in a thorough questionnaire about several topics, including their attitude to new drug prescribing, the number of visits by drug company representatives, the use of information sources on pharmacotherapy, and use of guidelines. Patients provided information about ten socio-demographic characteristics, including among other things age, gender, type of insurance, self-perceived health and highest level of education. The DNSGP-2 provided a unique opportunity to link detailed information on both GPs and patients to dispensing data from community pharmacies to obtain a complete overview of the Dutch primary care.

Pharmacy dispensing data are collected in the Netherlands by the Foundation for Pharmaceutical Statistics (SFK) [8]. Since 1990, the SFK has been collecting dispensing data from a growing number of community pharmacies in the Netherlands. In 2004, the catchment area of the SFK consisted of 1,540 community pharmacies, which represent 90% of the total number of Dutch pharmacies. The panel of pharmacies serves 13.5 million people and together dispense medicines, medical aid or bandages about 130 million times per year [8]. Both the DNSGP-2 and the SFK database display important -and partly overlapping- parts of Dutch primary care.

As in most other healthcare systems, information is increasingly stored in electronic form and made available for scientific research. Combining the different databases makes it possible to eliminate the shortcomings of individual databases and could result in opportunities greater than those presently thought of. Primary non-compliance, generic substitution, and interventions by pharmacists are just a few topics that could be addressed [13-15]. Therefore, the objective of this study was to determine the success rate of linkage of records of patients from the SFK dispensing database to the prescribing database of the DNSGP-2. Combining the information from both data sources offers an opportunity to gain more insight into the factors influencing drug exposure in patients.

## Methods

### Data collection

All GPs who participated in the DNSGP-2 listed the pharmacies where most of their patients filled their prescriptions. All pharmacists from the identified pharmacies were invited by letter to participate and followed-up with a telephone call 1–2 weeks later. To maximise the likelihood of tracing all patients we also contacted the pharmacies in the adjoining postal code areas. From the pharmacies that agreed to participate, dispensing data were collected from the SFK. The collected dispensing data covered the period January 1999 until December 2003, whereas the prescribing data were mainly from the year 2001. Since not all patient of the same GP visit the same pharmacy and not all pharmacies agreed to participate, we estimated the total number of eligible patients to be approximately 170,000. The estimation was based on the GP practice size, number of participating pharmacies and calculated by using estimates made by pharmacists of the percentage of a particular GP practice population that fills prescriptions at their pharmacy.

### Matching procedure

**Figure 1:**
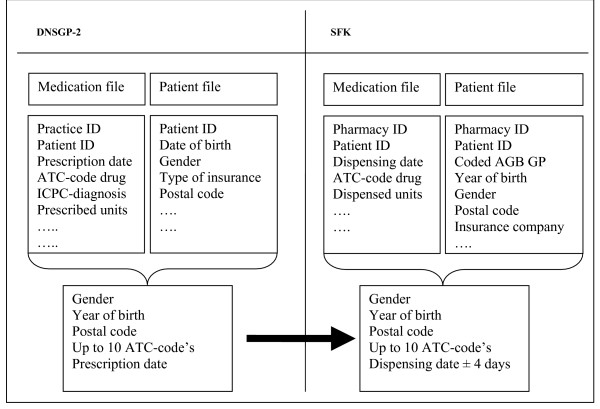
Structure of the matching process.

We used a deterministic linkage method to match patient records from both data sources by using patient identifiers year of birth, gender and 4-digit postal code. The combination of these three characteristics, however, is not unique enough to identify patients within a GP practice of approximately 2,300 patients, let alone in a Dutch pharmacy listing on average 9,000 patients [8]. Therefore, we used prescriptions characteristics as identifiers, namely the Anatomical Therapeutic Classification code (ATC-code) [16] and prescribing date. The identifiers are listed in Figure 1. The records from the prescription database of the DNSGP-2 were defined as enquiry records (i.e. those from which is searched), whereas the pharmacy records were defined as the file records (i.e. those that have to be retrieved). For each enquiry record, all file records are compared with respect to characteristics that are present in or logically related to both types of records.

The matching procedure consisted of several subsequent steps. Firstly, patients were blocked on a combination of gender, year of birth and the 4-digit numbers of their postal code. Subsequently, the prescriptions of the patients recorded in both databases were compared within the blocks. Prescriptions were matched on date, ATC-code and specificity of ATC-code. Up to ten prescriptions per patient were used for matching. A successful match of patients' records was defined as a positive match on gender, year of birth, postal code and a minimum of 50% of enquiry prescriptions found.

By using prescriptions as patient identifiers, several aspects have to be taken into account. Firstly, medication is not always filled on the same day it is prescribed. Certainly nowadays, patients frequently request a refill prescription by phone, often pick it up at the pharmacy the next day [17]. This results in a lag period of a few days between prescribing and dispensing. We defined a lag-time of four days as realistic. This means that patients' records could still be linked when there was a four days difference between prescribing and dispensing date.

Secondly, the ATC-code of the prescribed drug does not have to be identical in the seven characters to the dispensed drug. Differences in ATC-code may be caused by interventions of community pharmacists to optimise pharmacotherapy or prevent possible adverse drug reactions [14,18]. To allow interventions by the pharmacists, matching occurred in two stages. Patients were first matched using the complete ATC-code. When successfully linked, the patients' records were deleted from the enquiry and file database. The remaining patient records were matched using the first three characters of the ATC-code.

Thirdly, some drugs are prescribed more than others are. To benefit from this frequency of prescribing we categorised drugs in the enquiry database in three groups, namely drugs that were prescribed less than 5,000 times, between 5,000 and 10,000 times and more than 10,0000 times. Patient records were first matched by using drugs that were prescribed the least, followed by more common drugs. To safeguard patient privacy the study was conducted under strict privacy regulation of the DNSGP-2 [9].

## Results

**Figure 2:**
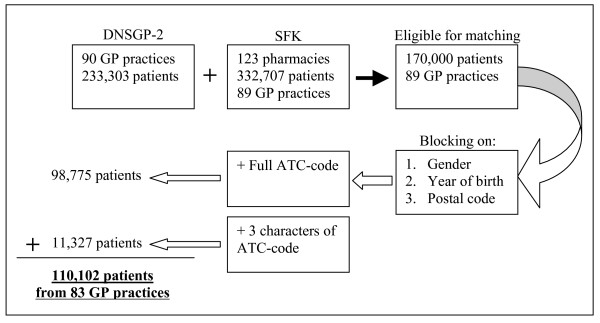
Sampling procedure and outcome of matching.

Among the 101 GP practices in the DNSGP-2, eleven were dispensing practices and were excluded. The analysis was based on 233,303 patients who received 1,841,271 prescriptions and were listed in 90 GP practices. Of the 203 community pharmacies approached, 123 (60.6%) pharmacies dispensed medication to patients of the particular GP and agreed to participate in the study. Of the 80 pharmacies that decided not to participate, 71 pharmacist responded never to dispense medication to patients of the DNSGP-2 GPs and nine pharmacies refused to participate in our study. This resulted in 89 GP practices of which both prescribing and dispensing data were available for matching. Taking into account the number of patients per GP practice, coverage of GP practices by pharmacies and registration period of both databases, we estimated that approximately 170,000 patients could in theory be traced in the pharmacy records. Figure 2 shows the sampling procedure and subsequent outcome of the different steps in the matching procedure.

Blocking patients on gender, year of birth, and postal code, matching on full 7-character ATC-code resulted in 98,775 (58.1%) uniquely identified patients. The remaining 71,225 patients not linked initially, were matched using the 3-character ATC-code of the medicines prescribed. Subsequently, 11,327 (6.7%) more patients were matched. In total, medical records of 110,102 (64.8%) patients from 83 GP practices were linked. Of these patients, virtually complete prescribing and dispensing histories are available. The prescribing data encompassed for most of the patients the year 2001, whereas the dispensing data covered the years 1999–2003.

The 110,102 linked patients filled 4,816,247 prescriptions of both GPs and medical specialists during the period 1999–2003, with an average of 43.7 (SD = 68.6) prescriptions per patient. Of these patients, 58.0% were female, 68.5% publicly insured and the mean age was 42.2 (SD = 21.8) years. Table 1 displays the characteristics of the DNSGP-2, SFK and linked database. Although statistically significant due to the large number of patients, there were no meaningful differences in patient characteristics between the original DNSGP-2 and linked dataset, except for the number of prescriptions per patient. The proportion of women ranged from 57.4% in the DNSGP-2 to 55.8% in the SFK data. The mean age of the patients in the linked-database was 42.2 years and higher than the mean age in the DNSGP-2 database (39.1 years) and the SFK-database (38.4 years). The number of prescriptions per patient in the SFK and linked database was higher than in the DNSGP-2 due to the longer registration period of the first two databases. After correction for the registration period, the number of prescriptions per patient per month was 0.68 for the DNSGP-2, 0.63 for the SFK, and 0.91 for the linked database. The higher number of prescriptions per patient in the linked database is the sum of prescriptions of GPs and medical specialists, whereas the DNSGP-2 only includes prescriptions of GPs. Furthermore, the higher number of prescriptions per patient is also the result of the fact that only patients with prescriptions could be linked. The characteristics of included GP practices in the linked-database were also comparable to the original sample of the DNSGP-2 with respect to type of office, situated in deprived areas, and urbanisation degree.

**Table 1:** Characteristics the patients and GP practices in the enquiry, field and final database

**Database**	**DNSGP-2**	**SFK**		
Type	Enquiry records	File records	Linked dataset	
Time period	Oct 2000 – Jun 2002	Jan 1999 – Dec 2003	Jan 1999 – Dec 2003	
% Female	57.8%	55.8%	58.0%	P < 0.02
% Publicly insured	67.5%	-*	68.5%	p < 0.00
Mean age (SD)	40.9 years (22.7)	38.4 years (22.7)	42.2 years (21.8)	p < 0.00
Mean number of Rx/patient (SD)	7.9 (11.9)	28.9 (59.6)	43.7 (68.6)	p < 0.00
Nr. of Rx/patients/month	0.68	0.63	0.91	
Nr. of patients	233,303	332,707	110,102	
Nr. of Rx	1,841,271	10,049,944	4,816,247	
Pharmacies	-**	123	112	
GP practice	N = 90***	N = 89	N = 83	
Solo	49 (54.4%)	46 (53.5%)	43 (51.8%)	p = 0.11
Duo	18 (20.0%)	17 (19.8%)	17 (20.5%)	
Group	14 (15.6%)	14 (16.3%)	14 (16.9%)	
Healthcare centre	9 (10.0%)	9 (10.5%)	9 (10.8%)	
Deprived area	8 (8.9%)	8 (8.3%)	8 (9.6%)	
Degree of urbanisation				
Not urbanised	9 (10.0%)	9 (10.5%)	9 (12.0%)	p = 0.21
Hardly urbanised	17 (18.9%)	16 (18.6%)	14 (16.9%)	
Averaged urbanised	19 (21.1%)	19 (22.1%)	19 (22.9%)	
Strongly urbanised	24 (26.7%)	22 (25.6%)	21 (24.1%)	
Extremely urbanised	21 (23.3%)	20 (23.3%)	20 (24.1%)	

We used chi-square to test for differences in % female, % publicly insured, practice type, and degree of urbanisation. T-test was used for mean age, and Mann-Whitney U-test for mean number of Rx/patient. Differences were assessed between DNSGP-2 versus linked dataset.

* Not used in the linkage process

** Not available in the DNSGP-2

*** Only non-dispensing GP practices included. In total 101 GP-practices collected prescribing data during the DNSGP-2

## Discussion

The primary objective of this study was to determine the success rate of linking dispensing data of community pharmacies to prescribing data of general practitioners. The completeness, quality and validity of patient characteristics play a vital role in record linkage processes [19,20]. If patient characteristics are not valid, or change over time, this could negatively influence the matching. Although gender and year of birth do not change over time, they are still susceptible to registration errors [21]. However, we assumed that pharmacies and GPs register the patient's date of birth accurately because it is often used to retrieve the patient's history from the computer systems. With respect to the completeness of the dispensing data Herings defined five scenarios leading to incompleteness, namely: (1) patients are not a member of the catchment area, but incidentally fill a prescription in the catchment area. (non-residents) (2) a patient number is not uniquely assigned to one patient (non-unique assignment) (3) a patient has more than one patient number in the register (internal multi-unique assignment) (4) a patient is registered in more than one pharmacy register (external multi-unique assignment) (5) a patient living in the catchment area fills a prescription outside the catchment area (non-eligible) [4].

In the linkage method we used, linkage of non-residents was unlikely. Although these patients filled a prescription in the catchment area, they most likely did not receive a prescription from a GP working in the catchment area. Non-unique assignment is also very unlikely in Dutch pharmacy and GP systems, as all systems do not allow multiple assignment of a patient ID. Internal multiple assignment of a patient to more than one number due to entry errors of e.g. date of birth, gender or postal code, is limited. Herings found in a sample of 2,000 patients that 1.4% of the patients were multiple coded in a pharmacy. Finally, dispensing data can also be incomplete when patients fill prescriptions outside the catchment area and thereby become non-eligible. Herings et al. estimated the completeness of drug dispensing histories in Dutch pharmacies [4]. They found that more than 99% of all patients had complete drug dispensing histories in cities where pharmacists maintained one central patient register.

The factor that had the most negative influence on the number of patients that could be matched was the different registration periods of the pharmacies. Most of the GP practices registered during twelve months (mean = 12, sd = 1.6) over the period October 2000 till January 2002 while participating in the DNSGP-2, but not all pharmacies registered the same complete twelve months. Missing months may be a main reason why patients' records could not be linked. If a drug was prescribed during a period in which no pharmacy data were available, this would lower the probability of linking. Another important factor that negatively influenced the matching process, are the pharmacies that refused to participate in the study. It is often difficult to trace patients back to a pharmacy, especially in larger cities where patients of a GP may visit different pharmacies. Part of the patients may go to one pharmacy, while the other part goes to another pharmacy. This means that if one pharmacy does not participate in the study a part of the patient records cannot be linked. In a sensitivity analysis we found that in isolated villages, the percentage of linked patients was higher. However, the lack of coverage of the GP practice by community pharmacies resulted probably only in a reduction of the number of patients that could be linked. We do not think this negatively affected the completeness of dispensing histories, because patients usually visit only one pharmacy in the Netherlands.

The final linkage percentage of 64.8% is dependent on several factors. One of the most important is the estimation of the catchment population. We estimated the total number of eligible patients to be 170,000, based on the number of patients per GP practice, coverage of GP practices by pharmacies and the registration period of both databases. The coverage of GP practices by pharmacies was calculated by using estimates made by pharmacists of the percentage of a particular GP practice population that fills prescriptions at their pharmacy. For the GP practices with less than 100% coverage, we performed a sensitivity analysis on the estimations of pharmacists of the proportion of patients from the GP practice filling prescriptions in their pharmacy. Taking into account a 10% error range, the catchment population ranged from 164,000 to 175,000 patients, meaning that the final linkage percentage ranged from 62.9% to 67.1%.

Furthermore, the final linkage percentage is also directly influenced by the definition of a successful match. We defined a successful match of patients' records when gender, year of birth, postal code and a minimum of 50% of enquiry prescriptions were positively matched. This cut-off value is, of course, arbitrary and lowering this requirement positively influences the percentage of patients being matched, but increases the number of false positives. Choosing 50% as a cut-off point, however, allows matching of records of patients who only received two prescriptions. Increasing the cut-off point to, for example 75%, excludes all patients who received up to four prescriptions of which one or more were changed.

Limitations of the linking process lie mainly in the available linkage keys. To ensure the privacy of patients, the SFK only collects the year of birth and not the complete date of birth. The combination of date of birth and gender is almost unique in a population of 2,000 individuals. Since the average Dutch GP practice consists of approximately 2,300 patients, the combination of gender and date of birth would almost be sufficient to identify patients within a GP practice. However, the specificity dramatically decreases for the combination of year of birth and gender.

We decided not to use type of insurance as a matching variable, because differentiating between private and public insurance was difficult as some pharmacies register both private and publicly insured patients of one insurance company using a single code. Finally, we did not validate our linkage procedure by contacting individual patients. This is under the privacy statements of the DNSGP-2 and the SFK prohibited. Several problems need further elaboration when linkage of both systems will be done on a continuous basis in the future.

## Conclusion

This study shows that linkage of dispensing and prescribing data is feasible with a combination of patient characteristics, such as gender, year of birth and postal code, and prescription characteristics, like prescription date and ATC-code. The final database contains both dispensing and prescribing data of medical specialists and GPs completed with detailed information on not only the patients, but also on community pharmacists and GPs. It offers an opportunity to gain insight into the magnitude and direction of forces directing drug utilisation in general practice.

## Competing interests

The author(s) declare that they have no competing interests.

## Authors' contributions

SF participated in the study design, carried out the programming of the data, and drafted the manuscript.

PS participated in the study design, carried out the programming of the data, and drafted the manuscript.

FG was involved in collecting the data and drafting the manuscript and revising it critically for important intellectual content

PG was involved in collecting the data, participated in the study design, was involved in drafting the manuscript and revising it critically for important intellectual content.

HL participated in the study design and was involved in drafting the manuscript and revising it critically for important intellectual content.

ER participated in the study design, was involved in drafting the manuscript and revising it critically for important intellectual content. ER had general supervision of the research group.

All authors read and approved the final manuscript.

## Pre-publication history

The pre-publication history for this paper can be accessed here:


